# Identification of parameters in routine blood and coagulation tests related to the severity of COVID-19

**DOI:** 10.7150/ijms.47494

**Published:** 2021-01-14

**Authors:** Rongrong Ding, Zongguo Yang, Dan Huang, Yanbing Wang, Xiufen Li, Xinlan Zhou, Li Yan, Wei Lu, Zhanqing Zhang

**Affiliations:** 1 Department of Hepatobiliary Medicine, Shanghai Public Health Clinical Center, Fudan University, Shanghai 201508, China.; 2 Department of Integrative Medicine, Shanghai Public Health Clinical Center, Fudan University, Shanghai 201508, China.

**Keywords:** Coronavirus disease 2019, COVID-19, highly sensitive C-reactive protein, FDP, fibrinogen degradation products

## Abstract

**Objective:** This study aimed to identify the predictive value of simple markers in routine blood and coagulation tests for the severity of coronavirus disease 2019 (COVID-19).

**Methods:** A total of 311 consecutive COVID-19 patients, including 281 patients with mild/moderate COVID-19 and 30 patients with severe/life-threatening COVID-19, were retrospectively enrolled. Logistic modeling and ROC curve analyses were used to assess the indexes for identifying disease severity.

**Results:** Lymphocyte and eosinophil counts of COVID-19 patients in the severe/life-threatening group were significantly lower than those of patients in the mild/moderate group (P < 0.001). Coagulation parameters, high-sensitivity C-reactive protein (hsCRP) levels and procalcitonin levels were higher in the severe/life-threatening group compared with the mild/moderate group (all P < 0.05). Univariate and multivariate logistic models revealed that hsCRP and fibrinogen degradation products (FDPs) were predictors of severe COVID-19 (OR = 1.072, P = 0.036; and OR = 1.831, P = 0.036, respectively). The AUROCs of hsCRP and FDP for predicting severe/life-threatening COVID-19 were 0.850 and 0.766, respectively. The optimal cutoffs of hsCRP and FDP for the severe/life-threatening type of COVID-19 were 22.41 mg/L and 0.95 µg/ml, respectively.

**Conclusion:** Serum CRP and FDP levels are positively related to the severity of COVID-19. This finding indicates that CRP and FDP levels may potentially be used as early predictors for severe illness and help physicians triage numerous patients in a short time.

## Introduction

An outbreak of coronavirus disease 2019 (COVID-19) caused by person-to-person transmission of severe acute respiratory syndrome coronavirus 2 (SARS-CoV-2) has spread rapidly worldwide with a spectrum of disease ranging from asymptomatic to critical illness 1. Globally, greater than 40 million cases of COVID-19 have now been reported to the WHO with greater than one million deaths 2. Given that COVID-19 patients with mild illness or asymptomatic carriers are prone to transmit the virus, the number of new and severe cases is increasing rapidly every day 3. Due to the genomic homologies of coronavirus pathogens, COVID-19 exhibits a similar clinical course and pathological features as severe acute respiratory syndrome (SARS) and Middle East respiratory syndrome (MERS) 4-6. Although most patients with COVID-19 are mild and can recover gradually after two weeks, approximately 15-20% of these patients develop severe illness 7,8. Moreover, patients with severe disease may quickly develop acute respiratory distress syndrome (ARDS), acute heart injury, secondary infection, and even multiple organ failure 1,6. Therapeutic measures, such as antiviral treatment, can contribute to alleviating severe cases of COVID-19 and improve the prognosis 9. Therefore, it is critically important to quickly and accurately recognize the severity of COVID-19 during the early stages of disease according to simple clinical parameters, so treatment can be initiated for severe cases in a timely manner.

Recently, various studies reported that laboratory changes, including hematologic parameters and coagulation indicators, correlated with poor prognosis in patients with severe pneumonia caused by SARS-CoV-2 10,11-13. Hematologic parameters, including leucocytes, neutrophil-to-lymphocyte ratio (NLR) and monocyte-to-lymphocyte ratio (MLR), have been reported as indicators in diagnosing and stratifying the risk of infectious diseases 14. A recent study indicated that the NLR may represent a reliable marker for evaluating the severity of COVID-19^15^. Moreover, coagulation dysfunction in COVID-19 patients is associated with an increased risk of death 16. SARS-CoV-2 may activate the innate immune system to clear the virus. However, an excessive immune response can cause an inflammatory storm, destroy microcirculation, activate the coagulation system and lead to disseminated intravascular coagulation (DIC) 17. Currently, some coagulation parameters, such as D-dimer and fibrin degradation product (FDP), have been described as prognostic factors of severe disease 10,12. However, studies on the predictive performances of hematologic parameters and coagulation markers in COVID-19 patients are on-going, and the clinical value of these markers in predicting severe illness needs to be further confirmed.

Therefore, in this retrospective study, we aimed to examine the correlation between hematologic parameters and coagulation markers and the severity of COVID-19. We also sought further identify any biomarkers that could serve as potential predictors of severe illness.

## Materials and methods

### Ethics statement

The study protocol and informed consent documents were reviewed and approved by the Ethics Committee of Shanghai Public Health Clinical Center, Fudan University (YJ-2020-S111-02). All COVID-19 patients provided written informed consent during admission.

### Patients

A total of 311 patients with COVID-9 confirmed by the SARS-CoV-2 nucleic acid test (RT-PCR) who were admitted to Shanghai Public Health Clinical Center, Fudan University from January 20 to March 15 were enrolled in our retrospective study. Clinical and laboratory data of the patients were collected from medical records. The laboratory data, including routine blood test parameters, high-sensitivity C-reactive protein (hsCRP) measurement, coagulation tests, and erythrocyte sedimentation rate (ESR), were investigated.

### Data collection

Routine blood test results; hsCRP; coagulation parameters, including APTT, FDP, fibrinogen (FIB), PT, international normalized ratio (INR), prothrombin time activity (PTA), and D-dimer; and ESR were determined on the first day after admission. All laboratory tests were conducted in the Department of Medical Laboratory, Shanghai Public Health Clinical Center, Fudan University.

### Definition

Two inflammatory predictors, including the monocyte-to-lymphocyte ratio (MLR) and neutrophil-to-lymphocyte ratio (NLR), were included in this analysis.

### Outcomes

Based on the 7^th^ trial version of the “Diagnosis and treatment program of novel coronavirus-infected pneumonia” released by the National Health Commission of the People's Republic of China 18, COVID-19 patients were divided into mild/moderate and severe/life-threatening groups. Patients meeting any of the following criteria were diagnosed with severe/life-threatening type: 1) polypnea with a respiratory rate ≥ 30 breaths per minute; 2) finger oxygen saturation ≤ 93% in a resting state; 3) PaO2/FiO2 ≤ 300 mmHg; 4) respiratory failure requiring mechanical ventilation; 5) emerging shock; and 6) combined with other organ failure requiring ICU care. Severe/life-threatening signs persisting for up to one week were considered severe disease.

### Statistical analysis

Statistical analysis was performed using the Statistical Package for the Social Sciences (SPSS version 22.0). The continuous variables are presented as the mean ± SD or median (range), which were compared using the independent *t* test or the Mann-Whitney *U* test, respectively. Categorical variables are presented as proportions and were compared using the chi-square test. Logistic regression models were used to assess the correlation between the laboratory parameters and disease severity. The area under the receiver operating characteristic curve (AUROC) and its 95% confidence interval (CI) were calculated to evaluate the prediction efficiency of the potential predictors. Decision curve analysis was conducted using the rmda package in the R program 19. A two-sided *P*<0.05 was considered statistically significant.

## Results

### Clinical characteristics

A total of 311 patients (162 males and 149 females) with COVID-19 were enrolled in the study. Among them, 281 (90.4%) patients were in the mild/moderate group, and 30 (9.6%) were in the severe/life-threatening group. The clinical characteristics of COVID-19 patients are presented in **Table 1**. The median age of all of the patients was 51 years (36-64 years). Patients with severe/life-threatening COVID-19 were significantly older than the patients with mild/moderate COVID-19 (median age, 64.5 years (56.3-71.2 years) vs 48 years (36-68 years), P < 0.001). Of the enrolled patients, 142 (50.53%) male patients were in the mild/moderate group, and 20 (66.67%) male patients were in the severe/life-threatening group. No significant differences in comorbidities were noted between the mild/moderate and severe/life-threatening groups. The most common initial signs and symptoms of the disease were fever (81.99%) and cough (54.66%). Less common symptoms included fatigue (19.94%), chest tightness (12.54%), headache and muscle pain (15.76%), diarrhea (7.72%), and nausea and loss of appetite (6.11%). In the severe/life-threatening group, 7 patients (23.33%) received noninvasive ventilation, 7 patients (23.77%) received invasive ventilation, and 6 patients (20.00%) received invasive ventilation and extracorporeal membrane oxygenation (ECMO). In the mild/moderate group, 228 patients (81.14%) received antiviral therapy, 83 patients (29.54%) received antibiotic therapy, 87 patients (30.96%) received treatment with thymosin alpha-1, and 34 patients (12.1%) received treatment with corticosteroids. In the severe/threatening group, all of the patients received antiviral and antibiotic therapy, and most of the patients received corticosteroid and thymosin alpha-1 therapy (93.33% and 96.67%, respectively). Significant differences in the number of patients who received antiviral therapy, antibiotic therapy, corticosteroids and thymosin alpha-1 were noted between the two groups (all P < 0.01). Five patients with life-threatening COVID-19 died.

### Routine blood tests and coagulation data

Inflammation and coagulation parameters on admission were compared between the mild/moderate and severe/life-threatening groups (**Table 2**). No significant differences in WBC counts, RBC counts, hemoglobin, monocyte counts, INR, PT or ESR were observed between the two groups (P > 0.05 for all). The blood classified counts of the severe/life-threatening patients exhibited an increase in neutrophils (median 3.54×10^9^/L vs. 2.88×10^9^/L, P = 0.005) and a decrease in lymphocytes (median 0.68 ×10^9^/L vs 1.15×10^9^/L, P < 0.001) and eosinophils (median 0 vs. 0.01×10^9^/L, P < 0.001). A similar difference was observed for platelet counts (median 146.0×10^9^/L vs 179.0×10^9^/L, P = 0.042). Compared with the mild/moderate group, the severe/life-threatening group exhibited higher D-dimer, APTT, FIB, and FDP levels (median 0.41 vs 0.91, 39.05 vs 42.40, 4.19 vs 4.72, and 0.92 vs 2.05, respectively). The indexes related to systemic inflammation, such as the NLR (median 5.33 vs 2.72), MLR (median 0.51 vs 0.36), hsCRP (median 50.88 vs 9.90) level, and procalcitonin (median 0.08 vs 0.02) level, increased significantly more in the severe/life-threatening COVID-19 group compared with the mild/moderate group.

### Predictive factors of COVID-19 severity

The predictive factors for the severe type of COVID-19 are presented in **Table 3**. According to univariate analysis, cardiovascular disease, old age, WBC counts, low lymphocytes, high neutrophils, low eosinophils, NLR, MLR, hsCRP, procalcitonin, D-dimer, PTA, APTT, FIB, FDP, antibiotic therapy, use of corticosteroids, and use of thymosin alpha-1 were significantly correlated with the severity of COVID-19 (all P<0.05). According to the multivariate analysis, only hsCRP and FDP were predictors of COVID-19 severity (OR = 1.072, 95% CI = 1.005 - 1.145, P = 0.036; OR = 1.831, 95% CI = 1.040 - 3.223, P = 0.0036, respectively).

### Predictive performances of serum inflammation and coagulation parameters for COVID-19 severity

**Figure 1:**
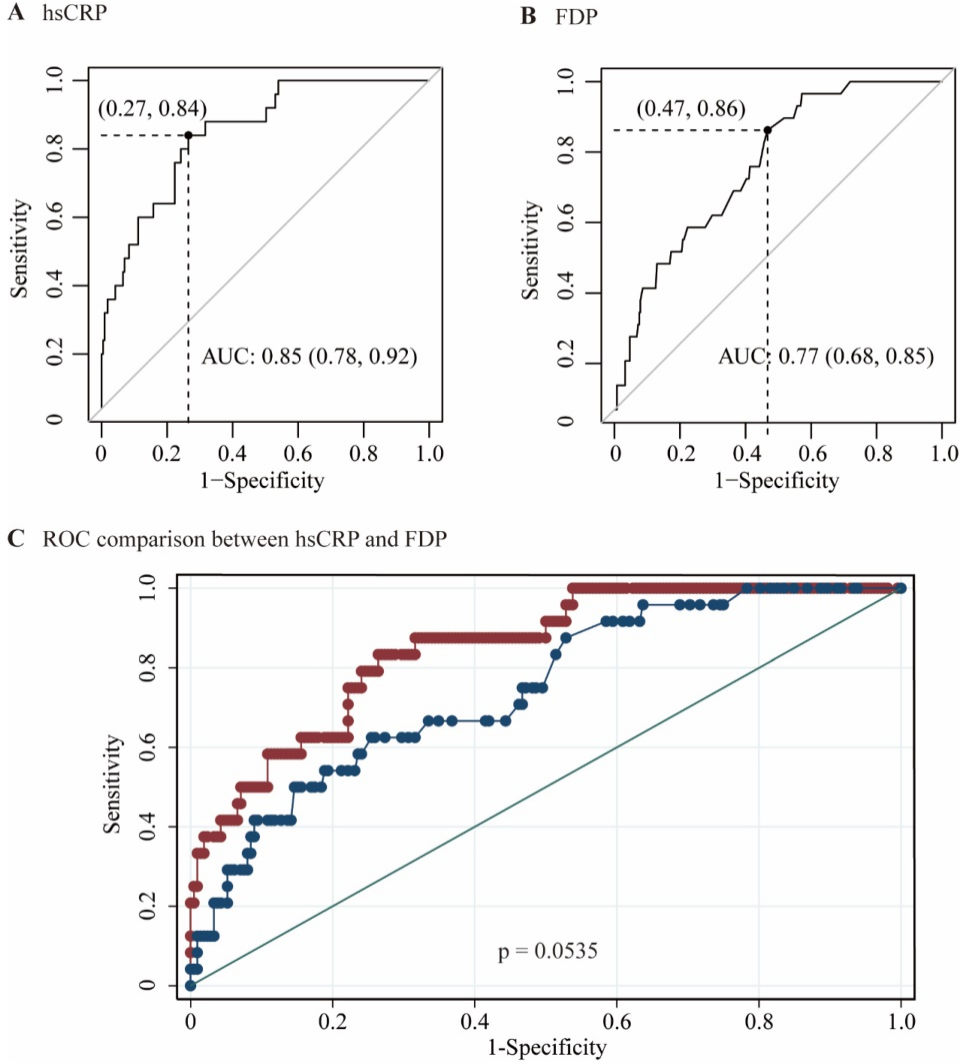
Area under receiver operating characteristic (AUROC) curves for the prediction of the severity of COVID-19 according to the diagnosis of severe / lift-threatening form as the reference. (A) AUROC of hsCRP; (B) AUROC of FDP; (C) AUROCs of hsCRP and FDP.

**Figure 2:**
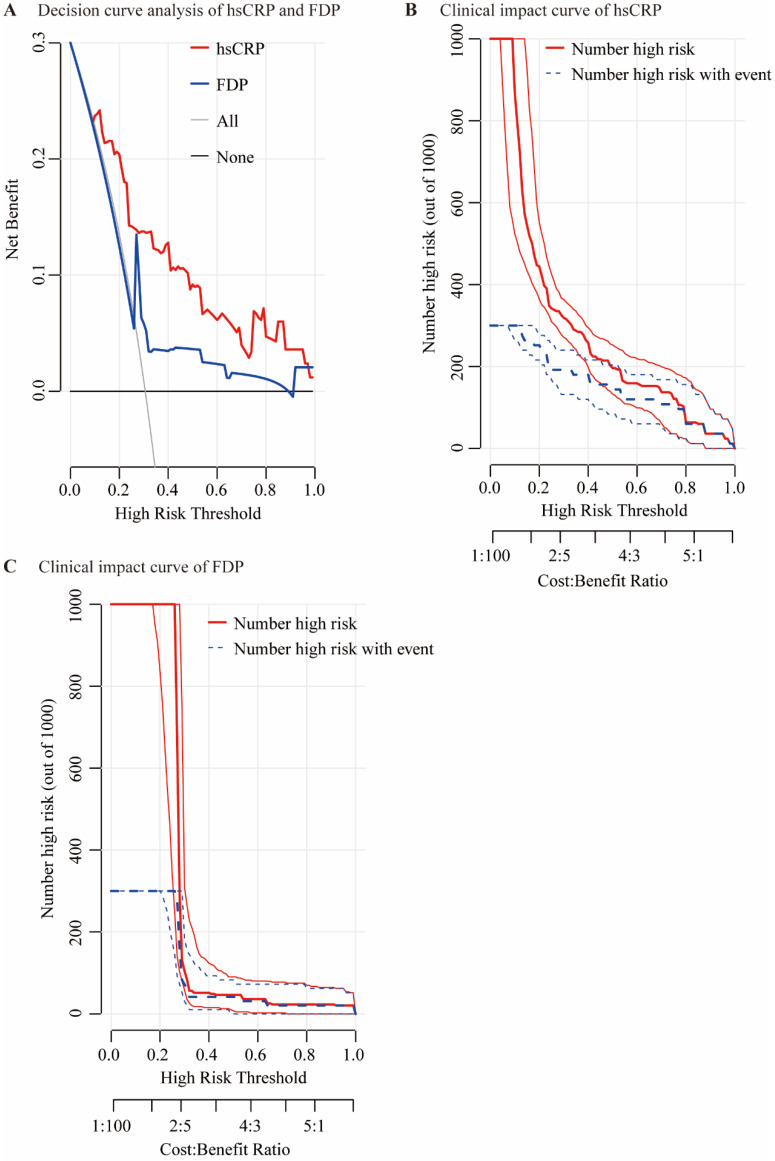
Decision curve analysis and clinical impact curves. Decision curve analysis for predicting the severity of COVID-19 of hsCRP and FDP (A); “None” refers to all the COVID-19 patients are considered as not having the outcome (severe / life-threatening form) and “All” to all the COVID-19 patients are considered as having the outcome. Clinical impact curves of hsCRP (B) and FDP (C); The number of high-risk patients and the number of high-risk patients with the outcome are plotted at different threshold probabilities within a given population.

The performances of hsCRP and FDP in predicting the severity of COVID-19 were calculated using ROC curves. The ROC curves of hsCRP and FDP are presented in **Figure 1**. The AUROCs of the indexes for predicting COVID-19 severity are shown in** Table 4**. The AUROCs of hsCRP and FDP for predicting severe/life-threatening COVID-19 were 0.850 (95% CI = 0.798-0.893) and 0.766 (95% CI = 0.714-0.812), respectively. Based on the estimated AUROCs to predict the severity of COVID-19, the performance of hsCRP versus FDP was comparable (P = 0.054). In addition, we conducted decision curve analysis to further investigate the clinical application values of the two markers. The decision curve analyses of hsCRP and FDP for predicting the severity of COVID-19 are presented in **Figure 2A**. The results showed that hsCRP exhibited the highest net benefit at any given threshold. Based on the above findings, we further plotted clinical impact curves to evaluate the clinical impact. The results demonstrated good predictive power of the two markers (**Figure 2B** and **C**).

### Predictive thresholds and accuracies of serum indexes for severe/life-threatening COVID-19

The predictive thresholds and accuracies of serum indexes for severe/life-threatening COVID-19 are presented in **Table 5**. Maximizing the sum of sensitivity and specificity, the optimal cutoffs of hsCRP and FDP were 22.41 mg/L and 0.95 µg/ml, respectively, for predicting the severe/life-threatening type of COVID-19. To obtain a sensitivity and specificity of at least 90%, the cutoffs of hsCRP were 9.85 mg/L and 46.42 mg/L, respectively, and the cutoffs of FDP were 0.78 µg/ml and 2.57 µg/ml, respectively.

## Discussion

The present study mainly demonstrated that hsCRP and FDP provided the greatest contribution to the prediction of critical illness by examining the results of hematologic parameters and coagulation function of COVID-19 patients.

Hematologic indexes, including lymphocyte, neutrophil, eosinophil counts, and NLR, helped to stratify COVID-19 patients. Our results corresponded to previous studies 20-22. Qin *et al.* 21 reported that compared to mild cases, severe cases tended to have lower lymphocyte counts, higher WBCs and NLRs, and lower percentages of monocytes, eosinophils and basophils in a cohort of 450 COVID-19 patients. A meta-analysis including 3377 COVID-19 patients showed that patients with severe and lethal illness had significantly higher WBC and lower lymphocyte and platelet counts 22. The NLR is typically used to identify bacterial infection severity and the prognosis of cardiovascular disease 23, 24. For COVID-19 patients, elevated NLR is an independent predictor for poor clinical outcome 15, 25. The combination of NLR and MLR potentially helped to predict severe COVID-19 with an AUROC of 0.93 26. In addition, SARS-CoV-2 causes an increase in inflammation, which leads to anemia owing to the destruction of RBCs and decreased erythrogenesis 26, 27. In contrast to these studies, no significant differences were noted between our groups with respect to RBC levels. Previous studies reported that COVID-19 patients with platelet counts of less than 100×10^9^/L only accounted for 5%; however, platelet counts of less than 150×10^9^/L were identified in 70-95% of patients with severe COVID-19 6, 7. Our findings were also consistent with those of these studies. Thus, thrombocytopenia in patients with COVID-19 did not seem to be a key predictor of COVID-19 progression 6, 28.

Elevated serum CRP levels in infected patients may be potential biomarkers for the diagnosis of infectious diseases 29. Currently, studies have reported that higher CRP levels are associated with adverse aspects of COVID-19, such as the development of ARDS, elevated troponin T levels, myocardial injury, and death 11, 30, 31. In our study, CRP levels in patients with severe/life-threatening COVID-19 were significantly increased compared with mild/moderate cases, which is consistent with earlier similar studies 32, 33. Furthermore, our study demonstrated very good performance of hsCRP in predicting the critical stage of COVID-19 with an AUROC of 0.85 and a cutoff level of 22.41 mg/L. In a large study that included 220 patients, the authors indicated that the AUROC of hsCRP for the early diagnosis of pneumonia complicated by sepsis was 0.82 with a cutoff of 55 mg/L 34. A study by Herold* et al.* 35 indicated that IL-6 and CRP highly predicted the need for invasive ventilation with maximal values > 80 pg/ml and > 97 mg/l, respectively. These results indicated that CRP could be used as a single tool to stratify COVID-19 patients. Clinically, elevated CRP levels may be an early indicator of nosocomial infection in patients with slow recovery of COVID-19 and may inform doctors to provide empirical antibiotic treatment as soon as possible to prevent deterioration of prognosis 7, 36.

Coagulation is a very organized process involving the interaction of endothelial cells, platelets and coagulation factors. Abnormal coagulation appears to be an important issue in COVID-19 patients. Recent data support that COVID-19 patients are at high risk of developing disseminated intravascular coagulation (DIC) or even death 7, 10. Higher levels of D-dimer and FDP levels are associated with poor prognosis in patients with COVID-19^10^. A meta-analysis reported that elevated fibrinogen and FDP levels on admission were associated with an increased risk of poor outcome in COVID-19 patients 37. The present study found that compared to patients with mild/moderate COVID-19, the PTA value in patients with severe/life-threatening illness was lower, whereas APTT, D-dimer, FDP, and FIB levels were higher. These results confirmed findings from earlier similar studies 10, 12. Additionally, our study indicated that FDP was a risk factor associated with the development of critical COVID-19; more importantly, we identified that the AUROC of the FDP level was 0.71 with a cutoff of 0.95 µg/ml. These findings strengthen the suggestion that routine monitoring of coagulation parameters may represent a potentially useful tool to improve the early diagnosis of critical COVID-19 and establish an accurate therapeutic strategy 38,39. It has been widely demonstrated that coagulation is activated and accelerated in response to infections because this mechanism may increase the physiological response 40,41. The mechanisms of coagulation dysfunction in COVID-19 are complex and potentially include direct injury of endothelial cells, imbalance of inflammatory response, over activation of immune system, ischemia-reperfusion injury, and drug factors 42.

This study has several limitations. First, because this study was a single-center and retrospective study, the characteristics of the enrolled patients may not be representative, and the findings should be validated in clinical studies with more power. Second, early treatment could help promote the recovery of patients with severe COVID-19. Future studies could further investigate the dynamic changes in in hsCRP and FDP in the treatment of patients with critical COVID-19 and the impacts of these markers on disease prognosis. Third, we did not examine the imaging index to assess the value of patients' imaging parameters and the dynamic changes in predicting severe/life-threatening cases. Finally, our study did not enroll pediatric patients. Previous studies have demonstrated that children of all ages can develop COVID-19. Compared with adult patients, pediatric patients tend to have a mild COVID-19 course with a good prognosis 43, 44.

In conclusion, CRP and FDP serum levels are positively related to the severity of COVID-19. This finding indicates that CRP and FDP may potentially be used as early predictors for severe illness, and this information may help physicians triage numerous patients in a short time.

## Figures and Tables

**Table 1:** Clinical Characteristics of patients infected with COVID-19

	All patients (n=311)	Mild/Ordinary (n=281)	Severe/life-threatening (n=30)	*P* value
Age (years), median (range)	51.0 (36.0-64.0)	48.0 (35.5-68.0)	64.5 (56.3-71.2)	<0.001
Male (n, %)	162 (52.09)	142 (50.53)	20 (66.67)	0.093
**Comorbidity**				
Hypertension (n, %)	79 (25.40)	68 (24.20)	11 (36.67)	0.136
Cardiovascular disease (n, %)	20 (6.43)	17 (6.04)	3 (10.00)	0.278
COPD (n, %)	7 (2.25)	5 (1.78)	2 (6.67)	0.086
Diabetes (n, %)	31 (9.97)	25 (8.89)	6 (20.00)	0.054
Chronic viral hepatitis (n, %)	9 (2.89)	7 (2.49)	2 (6.67)	0.195
Fatty liver disease (n, %)	24 (7.72)	22 (7.83)	2 (6.67)	0.821
**Signs and symptoms**				
Fever (n, %)	259 (81.99)	225 (80.07)	30 (100.00)	0.007
Cough (n, %)	170 (54.66)	150 (53.38)	20 (66.67)	0.165
Chest tightness (n, %)	39 (12.54)	18 (6.41)	21 (70.00)	<0.001
Nausea and loss of appetite (n, %)	19 (6.11)	16 (5.69)	3 (10.00)	0.410
Fatigue (n, %)	62 (19.94)	55 (19.57)	7 (23.33)	0.494
Diarrhoea (n, %)	24 (7.72)	24 (8.54)	0	---
Headache and muscle pain (n, %)	49 (15.76)	47 (16.73)	2 (6.67)	0.192
**Oxygen support**				
Nasal catheter oxygen (n, %)	94 (30.23)	84 (29.89)	10 (33.33)	0.697
Non-invasive ventilation orhigh-flow nasal cannula (n, %)	10 (3.22)	3 (1.07)	7 (23.33)	<0.001
Invasive mechanical ventilation (n, %)	7 (2.25)	0	7 (23.33)	---
Invasive mechanical ventilation and ECMO (n, %)	6 (1.93)	0	6 (20.00)	---
**Treatment strategies**				
Antiviral therapy	258 (82.96)	228 (81.14)	30 (100.00)	0.004
Antibiotic therapy	113 (36.33)	83 (29.54)	30 (100.00)	<0.001
Use of corticosteroid	62 (19.94)	34 (12.10)	28 (93.33)	<0.001
Use of thymosin alpha-1	116 (37.30)	87 (30.96)	29 (96.67)	<0.001
Death	5 (1.61)	0	5 (16.67)	---

COPD: chronic obstructive pulmonary diseases; ECMO: Extracorporeal membrane oxygenator.

**Table 2:** Levels of inflammation and coagulation parameters in patients with COVID-19 on admission

	All patients (n=311)	Mild/Ordinary (n=281)	Severe/Life-threatening (n=30)	*P* value
**Blood routine tests**				
WBC (×10^9^/L)	4.72 (3.89-5.97)	4.72 (3.91-5.92)	4.95 (3.74-7.64)	0.292
RBC (×10^12^/L)	4.46 (4.06-4.87)	4.45 (4.05-4.87)	4.59 (4.08-4.97)	0.462
Hemoglobin (g/L)	135.00 (125.75-147.00)	135.00 (126.00-147.00)	141.00 (123.25-148.25)	0.705
Neutrophil (×10^9^/L)	2.99 (2.39-4.01)	2.88 (2.35-3.90)	3.54 (2.83-5.29)	0.005
Lymphocyte (×10^9^/L)	1.12 (0.79-1.48)	1.14 (0.84-1.50)	0.68 (0.49-1.02)	<0.001
Eosinophils	0.01 (0.00-0.02)	0.01 (0.00-0.03)	0.00 (0.00-0.00)	<0.001
Platelet (×10^9^/L)	177.5 (143.0-221.0)	179.0 (143.2-223.7)	146.0 (120.7-201.7)	0.042
≥100 (n, %)	299 (96.14%)	271 (96.44%)	28 (93.33%)	0.947
80-100 (n, %)	5 (1.61%)	3 (1.07%)	2 (6.67%)	0.021
<80 (n, %)	7 (2.25%)	7 (2.49)	0	---
Monocyte (×10^9^/L)	0.42 (0.33-0.57)	0.42 (0.35-0.56)	0.29 (0.24-0.64)	0.081
**Coagulation parameters**				
INR	1.01 (0.97-1.05)	1.01 (0.97-1.04)	1.04 (0.97-1.10)	0.109
D-dimer (ug/ml)	0.43 (0.29-0.77)	0.41 (0.28-0.69)	0.91 (0.53-1.57)	<0.001
1-3 (n, %)	38 (12.22%)	28 (9.96%)	10 (33.33%)	<0.001
≥3 (n, %)	4 (1.28%)	0	4 (13.33%)	---
PT (second)	13.4 (13.00-13.80)	13.35 (13.00-13.80)	13.70 (12.90-14.00)	0.205
Prolongation of PT				
>6 (n, %)	1 (0.32%)	1 (0.35%)	0	---
3-6 (n, %)	2 (0.64%)	2 (0.71%)	0	---
<3 (n, %)	25 (8.04%)	20 (7.12%)	5 (16.67%)	0.071
PTA	99.0 (92.0-106.0)	99.0 (93.0-106.0)	93.0 (79.0-99.0)	0.001
APTT(second)	39.5 (36.2-42.5)	39.05 (36.10-41.92)	42.40 (38.45-46.00)	0.001
FIB (g/L)	4.26 (3.54-5.17)	4.19 (3.49-5.08)	4.72 (4.19-5.37)	0.009
FDP (µg/ml)	0.95 (0.48-1.76)	0.92 (0.42-1.54)	2.05 (1.06-4.20)	<0.001
**Other systemic inflammation parameters**				
NLR	2.84 (1.89-3.84)	2.72 (1.85-3.69)	5.33 (3.10-9.26)	<0.001
MLR	0.37 (0.28-0.54)	0.36 (0.27-0.52)	0.51 (0.33-0.65)	0.009
hsCRP (mg/L)	5.18 (11.07-30.80)	9.90 (4.60-23.58)	50.88 (25.05-83.25)	<0.001
Procalcitonin (ng/ml)	0.02 (0.03-0.05)	0.02 (0.02-0.05)	0.08 (0.06-0.15)	<0.001
ESR (mm/Hour)	27.75 (70.00-91.00)	70.00 (28.00-91.25)	68.00 (39.25-90.00)	0.628

WBC, white blood cells; INR, international normalized ratio; PT, prothrombin activity; PTA, prothrombin time activity; APTT, activated partial thromboplastin time; FIB, fibrinogen; FDP, fibrinogen degradation products; hsCRP, highly sensitivity C-reactive protein; ESR, erythrocyte sedimentation rate.

**Table 3:** Univariate and multivariate analyses of factors for the prediction of severe/life-threatening patients

Variables	Univariate analysis	*P*	Multivariate analysis	*P*
	OR (95% CI)		OR (95% CI)	
Hypertension, yes vs. no	1.813 (0.822-4.000)	0.140		
Cardiovascular disease, yes vs. no	4.726 (1.778-12.567)	**0.002**		
COPD, yes vs. no	3.943 (0.731-21.267)	0.111		
Diabetes, yes vs. no	2.560 (0.957-6.851)	0.061		
Chronicviral hepatitis, yes vs. no	2.796 (0.554-14.112)	0.213		
Fatty liver disease, yes vs. no	0.841 (0.188-9.765)	0.821		
Age, ≥ 60 vs.** <** 60 (years)	7.032 (3.006-16.451)	**< 0.001**		
WBC (×10^9^/L)	1.177 (1.042-1.329)	**0.009**		
RBC (×10^12^/L)	1.200 (0.611-2.356)	0.597		
Hemoglobin (g/L)	1.002 (0.978-1.027)	0.851		
Monocytes (×10^9^/L)	2.174 (0.436-10.830)	0.343		
Platelet (×10^9^/L)	0.995 (0.988-1.001)	0.126		
Lymphocyte, ≤ 0.5 vs. >0.5 (×10^9^/L)	50.545 (10.112-252.653)	**<0.001**		
Neutrophil, >6.3 vs. ≤ 6.3 (×10^9^/L)	6.251 (2.270-17.210)	**<0.001**		
Eosinophil <0.02 vs. ≥ 0.02 (×10^9^/L)	6.932 (1.614-29.775)	**0.009**		
NLR	1.269 (1.137-1.416)	**<0.001**		
MLR	12.017 (3.578-40.356)	**<0.001**		
hsCRP (mg/L)	1.045 (1.029-1.062)	**<0.001**	1.072 (1.005-1.145)	0.036
Procalcitonin (ng/ml)	11.034 (3.184-38.244)	**<0.001**		
ESR (mm/Hour)	1.002 (0.991-1.012)	0.747		
INR	3.293 (0.132-82.035)	0.468		
PT (second)	0.874 (0.666-1.147)	0.331		
PTA	0.956 (0.937-0.976)	**<0.001**		
APTT (second)	1.058 (1.008-1.110)	**0.023**		
FIB (g/L)	1.509 (1.127-2.020)	**0.006**		
FDP (ug/ml)	1.045 (1.005-1.088)	**0.029**	1.831 (1.040-3.223)	0.036
D-dimer, ≥1.0 vs. <1.0 (µg/ml)	7.687 (3.396-17.400)	**<0.001**		
Antiviral therapy, yes vs. no	6.741 (0.898-56.062)	0.064		
Antibiotic therapy, yes vs. no	69.181 (9.271-516.254)	**<0.001**		
Use of corticosteroid, yes vs. no	65.382 (18.817-277.285)	**<0.001**		
Use of thymosin alpha-1, yes vs. no	31.218 (7.274-133.978)	**<0.001**		

All the variables including WBC, neutrophils, lymphocytes, monocytes, platelet, NLR, MLR, hsCRP, procalcitonin, ESR, INR, D-dimer, PT, PTA, APTT, PTA,FIB, FDP were included in the univariate analysis as continuous data. Only variables with p < 0.05 in univariate model were included in the multivariate analysis. WBC, white blood cells; INR, international normalized ratio; PT, prothrombin activity; PTA, prothrombin time activity; APTT, activated partial thromboplastin time; FIB, fibrinogen; FDP, fibrin/fibrinogen degradation products; hsCRP, highly sensitivity C-reactive protein; ESR, erythrocyte sedimentation rate.

**Table 4:** Predictive performances of laboratory data for severe COVID-19

Indexes	AUROC	95%CI	P value
hsCRP (mg/L)	0.850	0.798-0.893	<0.0001
FDP (ug/ml)	0.766	0.798-0.893	<0.0001
**Comparison of AUROCs**			
hsCRP and FDP	P = 0.054		

**Table 5:** Predictive thresholds and Accuracies of Serum indexes for severe COVID-19

Indexes	Cutoff	Se (%)	Sp (%)	PPV (%)	NPV (%)	Accuracy
hsCRP (mg/L)	22.41	84.00	73.49	28.1	97.4	0.74
	9.85*	92.00	49.77	18.5	98.1	0.54
	46.42**	52.00	91.72	40.9	93.9	0.86
FDP (ug/ml)	0.95	86.21	53.24	18.6	96.9	0.57
	0.78*	93.10	44.24	17.1	98.1	0.49
	2.57**	41.38	91.37	37.2	92.7	0.86

Se, sensitivity; Sp, specificity; PPV, positive predictive value; NPV, negative predictive value. Cutoffs * were established by obtaining a sensitivity of at least 90%; Cutoffs ** were established by obtaining a specificity of at least 90%.
